# Crystal structure and Hirshfeld surface analysis of (*Z*)-4-chloro-*N*′-(4-oxo­thia­zol­idin-2-yl­idene)benzene­sulfono­hydrazide monohydrate

**DOI:** 10.1107/S2056989018013658

**Published:** 2018-10-12

**Authors:** Nikhila Pai, Sabine Foro, B. Thimme Gowda

**Affiliations:** a Department of Chemistry, Mangalore University, Mangalagangotri 574 199, Mangalore, India; b Institute of Materials Science, Darmstadt University of Technology, Alarich-Weiss-Str. 2, D-64287, Darmstadt, Germany; c Karnataka State Rural Development and Panchayat Raj University, Gadag 582 101, India

**Keywords:** crystal structure, thia­zole derivative, hydrogen bonds, Hirshfeld surface analysis

## Abstract

The asymmetric unit of the title thia­zole derivative containing a sulfonyl­hydrazinic moiety, C_9_H_8_ClN_3_O_3_S_2_·H_2_O, consists of two independent mol­ecules and two water mol­ecules. The central parts of the mol­ecules are twisted as both the mol­ecules are bent at both the S and N atoms. In the crystal, N—H⋯N, N—H⋯O, C—H⋯O and O—H⋯O hydrogen-bonding inter­actions connect the mol­ecules, forming layers parallel to the *ab* plane. Two-dimensional fingerprint plots associated with the Hirshfeld surface show that the largest contributions to the crystal packing come from O⋯H/H⋯O (32.9%) and H⋯H (22.6%) inter­actions.

## Abstract 2

The asymmetric unit contains two independent mol­ecules and two water mol­ecules. The central parts of both the mol­ecules are twisted as both mol­ecules are bent at both the S and N atoms. The crystal structure features N—H⋯N, N—H⋯O, C—H⋯O and O—H⋯O inter­molecular inter­actions. Two-dimensional fingerprint plots show that the largest contributions to the crystal stability come from O⋯H/H⋯O and H⋯H inter­actions.

## Chemical context   

Sulfonamides are of inter­est as this class of compounds exhib­its a wide array of biological activities such as anti­tumor, anti­bacterial, diuretic and hypoglycaemic activities (Kamal *et al.*, 2007 ▸). It has been reported that incorporation of hydrazine moieties increases the carbonic anhydrase inhibition activity (Winum *et al.*, 2005 ▸). Along with the sulfonamide group, the presence of the 2-hydrazino-thia­zole moiety enhances the pharmacological activities. The thiozoyl group is of inter­est because of its medicinal use in anti­tumor (Holla *et al.*, 2003 ▸; Kappe *et al.*, 2004 ▸), hyposensitive (Dash *et al.*, 1980 ▸), anti-HIV (Patt *et al.*, 1992 ▸), anti­microbial and anti­cancer agents (Frère *et al.*, 2003 ▸). Sulfonyl­hydrazines and their derivatives can easily be prepared and are stable. We report herein the synthesis and structure of the title compound, which is a new thia­zole compound containing a sulfonyl­hydrazinic moiety.
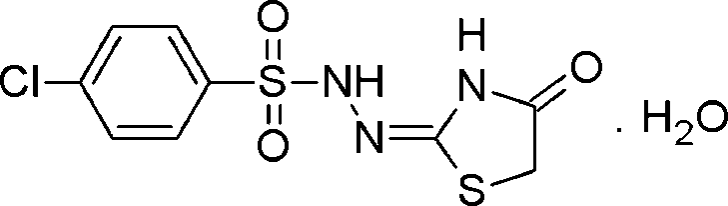


## Structural commentary   

**Figure 1:**
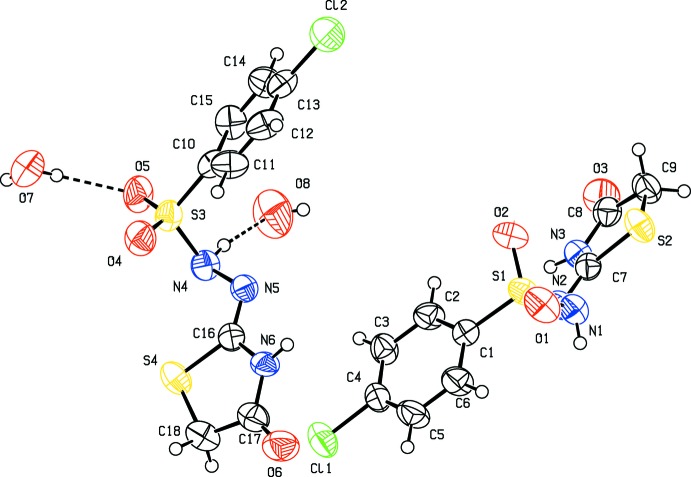
The mol­ecular structure of the title compound showing displacement ellipsoids at the 50% probability level.

The asymmetric unit of the title compound contains two independent mol­ecules and two water mol­ecules (Fig. 1 ▸). The C8—O3 and C17—O6 bond lengths of 1.202 (5) Å, 1.218 (6) Å, respectively, are consistent with C=O double-bond character. Similarly, the values of the C7—N2 and C16—N5 bond lengths [1.285 (5) and 1.276 (5) Å, respectively] are close to that of a typical C=N double bond, while the longer C7—N3 and C16—N6 bond lengths of 1.370 (5) and 1.381 (5) Å, respect­ively, are consistent with the normal C—N single bonds, indicating that the compound exists in the Schiff base form. Further, the N1—N2 and N4—N5 bond lengths of 1.440 (5) and 1.442 (5) Å, respectively, and the S1—N1 and S3—N4 bond lengths of 1.644 (4) and 1.649 (4) Å, respectively, are in agreement with single-bond character.

The central parts of both mol­ecules are twisted as they are bent at the S (S1 and S3) and N (N2 and N5) atoms as indicated by the C1—S1—N1—N2 and S1—N1—N2—C7 torsion angles of 57.0 (3) and 111.8 (3)°, respectively, and by the C10—S3—N4—N5 and S3—N4—N5—C16 torsion angles of 57.6 (3) and 109.7 (3)°, respectively. The sulfonyl­hydrazide bond exists in the synclinal conformation preferred by aromatic sulfonamides (Purandara *et al.*, 2017 ▸), with C—S—N—N torsion angles of 57.0 (3) and 57.6 (3)° in the two independent mol­ecules. The geometrical parameters for the thia­zole and benzene rings are within the expected ranges and comparable with those of other substituted thia­zoles or benzene­sulfonyl­hydrazide derivatives (Zaharia *et al.*, 2010 ▸). The C7—S2—C9 and C16—S4—C18 angles in the two mol­ecules have the same value of 91.4 (2)°, and it is similar to the angles typically observed in thia­zole derivatives (Form *et al.*, 1974 ▸). The thia­zole rings are approximately planar (r.m.s. deviations of 0.011 and 0.029 Å for S2/N3/C7–C9 and S4/N6/C16–C18, respectively), and form dihedral angles of 26.18 (15) and 37.19 (12)° with the aromatic ring of the *p*-chloro­phenyl­sulfonyl groups.

## Supra­molecular features   

**Figure 2:**
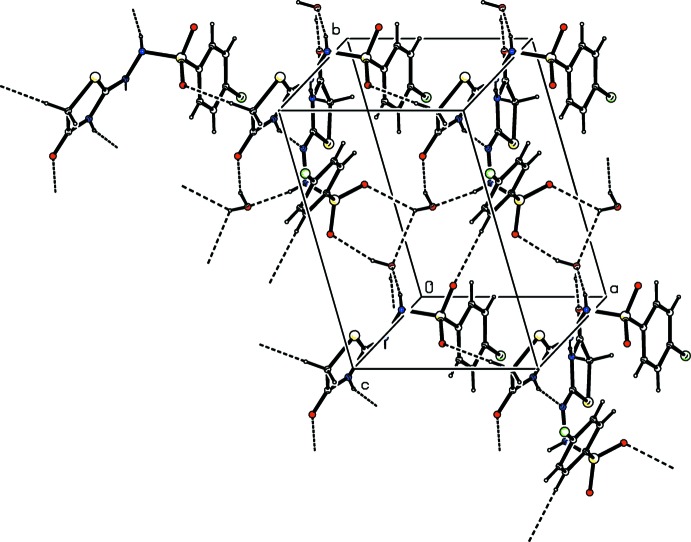
The mol­ecular packing of the title compound, with hydrogen bonds (Table 1 ▸) shown as dashed lines.

In the crystal, the two independent mol­ecules are linked into dimers by pairs of N—H⋯N hydrogen bonds, forming rings with an 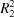
(8) graph-set motif. These dimers are connected by C—H⋯O hydrogen bonds involving the thia­zole C—H and a sulfonyl O atom into chains running parallel to the *a* axis (Table 1 ▸, Fig. 2 ▸). The water mol­ecules are involved both in the enforcement of the dimers through N—H⋯O and O—H⋯O hydrogen bonds, forming 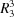
(9) rings, and in inter-chain O—H⋯O hydrogen-bonding inter­actions, forming layers parallel to the *ab* plane.

## Database survey   

Although a search in the Cambridge. Structural Database (CSD, Version 5.39, update of August 2018; Groom *et al.*, 2016 ▸) revealed several reports of the crystal structure of sulfonamides and thia­zole (Gowda *et al.*, 2008 ▸, 2009 ▸), there are only a few reports on the crystal structures of sulfonyl­hydrazides functionalized by thia­zole groups (Zaharia *et al.*, 2010 ▸). Comparison of the structure of the title compound with that of *N*′-(5-acetyl-4-methyl-4,5-di­hydro­thia­zol-2-yl)benz­ene­sulfono­hydrazide (Zaharia *et al.*, 2010 ▸) indicates that the electron-withdrawing chloro group does not impart sufficient inductive effect to reduce the electron density on the benzene ring, and that the ability of the aromatic C—H groups to participate in C—H⋯O inter­actions is very much reduced. Partial double-bond character is observed between the hydrazinyl N atom and the adjacent benzo­thia­zole moiety in 2-[2-(3-nitro­benzene­sulfon­yl)hydrazin­yl]-1,3-benzo­thia­zole (Morscher *et al.*, 2018 ▸). The orientation of the thia­zole ring in the title compound is similar to that of (*Z*)-methyl 2-[(*Z*)-4-oxo-2-(2-tosyl­hydrazono)thia­zolidin-5-yl­idene]acetate and (*Z*)-methyl-2-[(*Z*)-2-(ethyl­imino)-4-oxo-3-(phenyl­amino)­thia­zolidin-5-yl­idene]acetate (Hassan *et al.*, 2016 ▸). The mol­ecule of *N*′-{3-[3-(tri­fluoro­meth­yl)phen­yl]-1,3-thia­zol-2(3*H*)-yl­idene}benzene­sulfono­hydrazide (Chen *et al.*, 2015 ▸) is observed to have a Schiff base conformation.

## Hirshfeld Surface Analysis   

**Figure 3:**
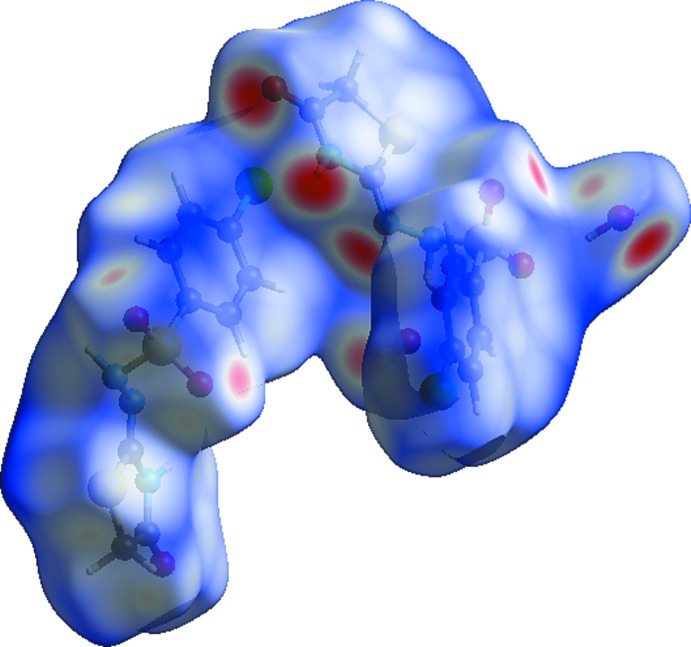
View of the Hirshfeld surface mapped over *d*_norm_.

**Figure 4:**
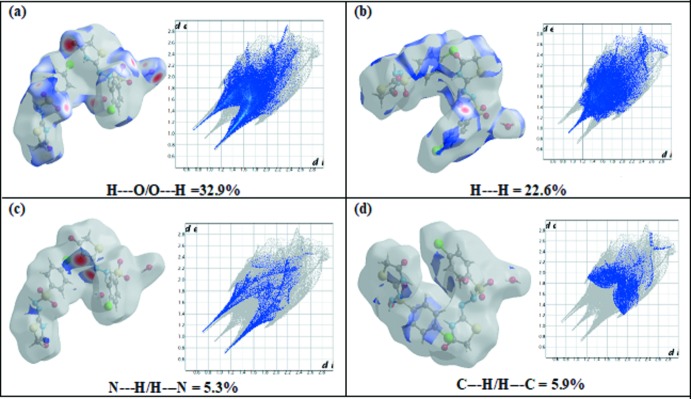
The two dimensional fingerprint (FP) plot for the title compound, delineated into (*a*) O⋯H/H⋯O, (*b*) H⋯H, (*c*) N⋯H/H⋯N and (*d*) C⋯H/H⋯C inter­actions. *d*_norm_ surfaces for each plot indicating the relevant surface patches associated with the specific contacts are shown on the left.

**Figure 5:**
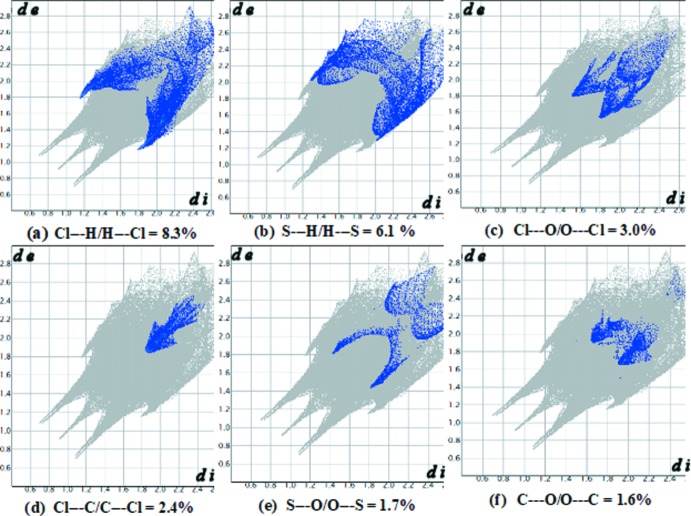
Fingerprint plots of inter­actions, listing their percentage contributions: (*a*) Cl⋯H/H⋯Cl, (*b*) S⋯H/H⋯S, (*c*) Cl⋯O/O⋯Cl, (*d*) Cl⋯C/C⋯Cl, (*e*) S⋯O/O⋯S and (*f*) C⋯O/O⋯C.

In order to explore the role of weak inter­molecular inter­actions in the crystal packing, Hirshfeld surfaces (*d*_norm_) and related fingerprint plots were generated using *CrystalExplorer17.5* (McKinnon *et al.*, 2007 ▸; Spackman *et al.*, 2008 ▸; Spackman & Jayatilaka, 2009 ▸; Wolff *et al.*, 2012 ▸). The three-dimensional mol­ecular Hirshfeld surfaces were generated using a high standard surface resolution over a colour scale of −0.6355 to 1.5137 a.u. for *d*_norm_. To identify the normalized contacts, the *d*_norm_ function is used, which is expressed as; *d*_norm_ = (*d*_i_ − *r*_i_^vdw^)/*r*_i_^vdw^ + (*d*_e_ − *r*_e_^vdw^)/*r*_e_^vdw^ (Shit *et al.*, 2016 ▸), where *d*_i_ and *d*_e_ are the distances from inter­nal and external atoms to the Hirshfeld surface and *r*_i_^vdw^ and *r*_e_^vdw^ are the van der Waals radii of the atoms inside and outside the surface. On the Hirshfeld surfaces mapped over *d*_norm_ (Fig. 3 ▸), strong N—H⋯N and S—O⋯H inter­actions are observed as red spots close to atoms N5, N6 and O6. Furthermore, the two-dimensional fingerprint plots indicate that the largest contributions are from O⋯H/H⋯O contacts, which contribute 32.9% to the Hirshfeld surface (Fig. 4 ▸ *a*) with *d*_i_ + *d*_e_ ∼ 1.9 Å. The presence of water mol­ecules in the unit cell provides the largest contribution to the stability of the crystal packing. The next largest contrib­utor is from H⋯H inter­actions, which contribute 22.6%. A single sharp spike can be seen in the middle region of the plot, at *d*_i_ = *d*_e_ = 0.9 Å (Fig. 4 ▸ *b*). The N⋯H contacts, which refer to N—H⋯N inter­actions, contribute 5.3% to the surface. Two sharp spikes having *d*_i_ + *d*_e_ = 1.8 Å (Fig. 4 ▸ *c*) are observed. The C⋯H contacts contribute 5.9% to the Hirshfeld surface, featuring a wide region with *d*_i_ + *d*_e_ = 3.1 Å (Fig. 4 ▸ *d*). The different inter­atomic contacts and percentage contributions to the Hirshfeld surface are Cl⋯H/H⋯Cl (8.3%), S⋯H/H⋯S (6.1%), Cl⋯O/O⋯Cl (3.0%), Cl⋯C/C⋯Cl (2.4%), S⋯O/O⋯S (1.7%), and C⋯O/O⋯C (1.6%) as depicted in the fingerprint plots (Fig. 5 ▸ *a*–*f*).

## Synthesis and crystallization   

4-Chloro-*N*′-(4-oxo-4,5-di­hydro-1,3-thia­zol-2-yl)benzene-1-sulfono­hydrazide was prepared by adding 4-chloro benzene­sulfonyl chloride (0.02 mol) under stirring to a solution of thio­semicarbazide (0.02 mol) in 5% aqueous NaOH solution (20 ml). The reaction mixture was stirred at room temperature for 1 h, then diluted twofold with water and neutralized with glacial acetic acid. The solid 2-(4-chloro­benzene-1-sulfon­yl)hydrazine-1-carbo­thio­amide (*A*) obtained was crystallized from acetic acid. Mono­chloro­acetic acid (0.01 mol) and anhydrous sodium acetate (0.04 mol) were added to *A* (0.01 mol) in glacial acetic acid. The reaction mixture was refluxed for 8–10 h and the completion of the reaction was checked by TLC. The reaction mixture was then poured into cold water. The resulted precipitate of the title compound was separated by vacuum filtration. Prismatic colourless single crystals of the title compound were grown from a mixture of aceto­nitrile-DMF (5:1 *v*/*v*) by slow evaporation of the solvent. The purity of the compound was checked by TLC and characterized by IR spectroscopy. The characteristic IR absorptions observed at 3095.9, 1639.5, 1458.7, 1343.2, 1139.4, and 1215.7 cm^−1^ correspond to N—H, C=O, C=N, S=O asymmetric and symmetric, and C—S absorptions, respectively. The ^1^H and ^13^C spectra of the title compound are as follows: ^1^H (400MHz, DMSO-*d*_6_); δ 3.45 (*d*, 2H, –CH_2_), 7.68–7.86 (*m*, 4H, Ar—H), 10.01 (*s*, 1H), 11.96 (*s*, 1H). ^13^C NMR (400 MHz, DMSO-*d*_6_); δ 36.8, 128.4, 129.1, 131.1,132.5, 133.9, 137.2, 165.4, 185.5.

## Refinement   

Crystal data, data collection and structure refinement details are summarized in Table 2 ▸. H atoms bonded to C were positioned with idealized geometry using a riding model with C—H = 0.93 Å (aromatic) or 0.97 Å (methyl­ene). The H atoms of the NH groups and the H atoms of the water mol­ecules were located in a difference-Fourier map and later refined with the N—H and O—H bond lengths constrained to be 0.86 (2) and 0.82 (2) Å, respectively. All H atoms were refined with isotropic displacement parameters set at 1.2*U*_eq_ of the parent atom.

## Supplementary Material

Crystal structure: contains datablock(s) I, global. DOI: 10.1107/S2056989018013658/rz5243sup1.cif

Structure factors: contains datablock(s) I. DOI: 10.1107/S2056989018013658/rz5243Isup2.hkl

Supporting information file. DOI: 10.1107/S2056989018013658/rz5243Isup3.cml

CCDC reference: 1869597

Additional supporting information:  crystallographic information; 3D view; checkCIF report

## Figures and Tables

**Table 1:** Hydrogen-bond geometry (Å, °)

*D*—H⋯*A*	*D*—H	H⋯*A*	*D*⋯*A*	*D*—H⋯*A*
N1—H1*N*⋯O7^i^	0.84 (2)	2.07 (2)	2.900 (6)	168 (4)
N3—H3*N*⋯N5^ii^	0.85 (2)	2.07 (2)	2.895 (5)	162 (4)
C9—H9*B*⋯O2^ii^	0.97	2.42	3.236 (6)	141
N4—H4*N*⋯O8	0.85 (2)	1.95 (2)	2.788 (6)	168 (4)
N6—H6*N*⋯N2^iii^	0.85 (2)	1.97 (2)	2.808 (5)	170 (4)
C15—H15⋯O1^iv^	0.93	2.55	3.355 (5)	145
O7—H71⋯O5	0.82 (2)	2.08 (3)	2.868 (5)	162 (6)
O7—H72⋯O6^iv^	0.82 (2)	1.99 (2)	2.810 (5)	174 (6)
O8—H81⋯O4^ii^	0.82 (2)	2.35 (6)	2.987 (6)	136 (7)
O8—H81⋯O7^ii^	0.82 (2)	2.50 (7)	3.034 (7)	124 (7)
O8—H82⋯O3^iii^	0.83 (2)	2.37 (3)	3.159 (7)	159 (8)

Symmetry codes: (i) 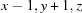; (ii) 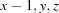; (iii) 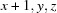; (iv) 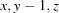.

**Table 2:** Experimental details

Crystal data	
Chemical formula	C_9_H_8_ClN_3_O_3_S_2_·H_2_O
*M*_r_	323.77
Crystal system, space group	Triclinic, *P* 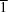
Temperature (K)	293
*a*, *b*, *c* (Å)	7.6276 (6), 11.090 (1), 17.116 (2)
α, β, γ (°)	96.95 (1), 99.49 (1), 106.08 (1)
*V* (Å^3^)	1350.8 (2)
*Z*	4
Radiation type	Mo *K*α
μ (mm^−1^)	0.60
Crystal size (mm)	0.42 × 0.20 × 0.06

Data collection	
Diffractometer	Oxford Diffraction Xcalibur Single Crystal X-ray diffractometer with a Sapphire CCD detector
Absorption correction	Multi-scan (*CrysAlis RED*; Oxford Diffraction, 2009 ▸)
*T*_min_, *T*_max_	0.785, 0.965
No. of measured, independent and observed [*I* > 2σ(*I*)] reflections	8631, 4935, 3375
*R*_int_	0.026
(sin θ/λ)_max_ (Å^−1^)	0.602

Refinement	
*R*[*F* ^2^ > 2σ(*F* ^2^)], *wR*(*F* ^2^), *S*	0.058, 0.161, 1.05
No. of reflections	4935
No. of parameters	367
No. of restraints	8
H-atom treatment	H atoms treated by a mixture of independent and constrained refinement
Δρ_max_, Δρ_min_ (e Å^−3^)	0.52, −0.25

Computer programs: *CrysAlis CCD* and *CrysAlis RED* (Oxford Diffraction, 2009 ▸), *SHELXS2013* (Sheldrick, 2008 ▸), *SHELXL2014* (Sheldrick, 2015 ▸) and *PLATON* (Spek, 2009 ▸).

## supplementary crystallographic information

### Crystal data

**Table:**

C_9_H_8_ClN_3_O_3_S_2_·H_2_O	*Z* = 4
*M**_r_* = 323.77	*F*(000) = 664
Triclinic, *P*1	*D*_x_ = 1.592 Mg m^−^^3^
*a* = 7.6276 (6) Å	Mo *K*α radiation, λ = 0.71073 Å
*b* = 11.090 (1) Å	Cell parameters from 2682 reflections
*c* = 17.116 (2) Å	θ = 2.8–27.8°
α = 96.95 (1)°	µ = 0.60 mm^−^^1^
β = 99.49 (1)°	*T* = 293 K
γ = 106.08 (1)°	Prism, colourless
*V* = 1350.8 (2) Å^3^	0.42 × 0.20 × 0.06 mm

### Data collection

**Table:**

Oxford Diffraction Xcalibur Single Crystal X-ray diffractometer with a Sapphire CCD detector	3375 reflections with *I* > 2σ(*I*)
Radiation source: Enhance (Mo) X-ray Source	*R*_int_ = 0.026
Rotation method data acquisition using ω scans	θ_max_ = 25.4°, θ_min_ = 2.8°
Absorption correction: multi-scan (CrysAlis RED; Oxford Diffraction, 2009)	*h* = −9→5
*T*_min_ = 0.785, *T*_max_ = 0.965	*k* = −13→13
8631 measured reflections	*l* = −20→20
4935 independent reflections	

### Refinement

**Table:**

Refinement on *F*^2^	8 restraints
Least-squares matrix: full	Hydrogen site location: mixed
*R*[*F*^2^ > 2σ(*F*^2^)] = 0.058	H atoms treated by a mixture of independent and constrained refinement
*wR*(*F*^2^) = 0.161	*w* = 1/[σ^2^(*F*_o_^2^) + (0.0767*P*)^2^ + 1.1929*P*] where *P* = (*F*_o_^2^ + 2*F*_c_^2^)/3
*S* = 1.05	(Δ/σ)_max_ = 0.001
4935 reflections	Δρ_max_ = 0.52 e Å^−^^3^
367 parameters	Δρ_min_ = −0.25 e Å^−^^3^

### Special details

**Table:**

Geometry. All esds (except the esd in the dihedral angle between two l.s. planes) are estimated using the full covariance matrix. The cell esds are taken into account individually in the estimation of esds in distances, angles and torsion angles; correlations between esds in cell parameters are only used when they are defined by crystal symmetry. An approximate (isotropic) treatment of cell esds is used for estimating esds involving l.s. planes.

### Fractional atomic coordinates and isotropic or equivalent isotropic displacement parameters (Å^2^)

**Table:**

	*x*	*y*	*z*	*U*_iso_*/*U*_eq_	
Cl1	0.33684 (17)	0.77231 (13)	0.00096 (7)	0.0689 (4)	
S1	0.23228 (14)	1.01774 (10)	0.33163 (6)	0.0479 (3)	
S2	−0.21540 (16)	0.96967 (12)	0.42899 (7)	0.0594 (3)	
O1	0.3605 (4)	1.1427 (3)	0.35513 (19)	0.0659 (9)	
O2	0.2255 (4)	0.9268 (3)	0.38508 (18)	0.0626 (8)	
O3	−0.6004 (5)	0.6450 (3)	0.3600 (2)	0.0732 (10)	
N1	0.0248 (5)	1.0382 (3)	0.3165 (2)	0.0484 (8)	
H1N	0.024 (6)	1.086 (4)	0.282 (2)	0.058*	
N2	−0.1246 (4)	0.9225 (3)	0.2827 (2)	0.0471 (8)	
N3	−0.3702 (4)	0.7765 (3)	0.3134 (2)	0.0460 (8)	
H3N	−0.380 (6)	0.721 (3)	0.2726 (19)	0.055*	
C1	0.2676 (5)	0.9482 (4)	0.2392 (2)	0.0421 (9)	
C2	0.2102 (6)	0.8168 (4)	0.2167 (3)	0.0515 (10)	
H2	0.1557	0.7651	0.2505	0.062*	
C3	0.2345 (6)	0.7627 (4)	0.1432 (3)	0.0523 (11)	
H3	0.1967	0.6747	0.1273	0.063*	
C4	0.3146 (5)	0.8409 (4)	0.0946 (2)	0.0475 (10)	
C5	0.3755 (7)	0.9718 (5)	0.1168 (3)	0.0617 (12)	
H5	0.4315	1.0231	0.0831	0.074*	
C6	0.3514 (7)	1.0250 (4)	0.1899 (3)	0.0577 (12)	
H6	0.3920	1.1131	0.2060	0.069*	
C7	−0.2275 (5)	0.8889 (4)	0.3335 (2)	0.0419 (9)	
C8	−0.4741 (6)	0.7422 (5)	0.3700 (3)	0.0522 (11)	
C9	−0.4074 (6)	0.8429 (5)	0.4445 (3)	0.0600 (12)	
H9A	−0.3669	0.8068	0.4905	0.072*	
H9B	−0.5080	0.8761	0.4549	0.072*	
Cl2	0.8418 (2)	0.63805 (14)	0.57661 (8)	0.0805 (4)	
S3	0.78365 (15)	0.43436 (10)	0.21411 (7)	0.0511 (3)	
S4	0.74715 (17)	0.59857 (12)	0.02143 (7)	0.0603 (3)	
O4	0.9602 (4)	0.4892 (3)	0.1948 (2)	0.0670 (9)	
O5	0.7003 (5)	0.2980 (3)	0.1978 (2)	0.0691 (9)	
O6	0.8569 (5)	0.9655 (4)	0.0519 (2)	0.0762 (10)	
N4	0.6297 (5)	0.4853 (3)	0.1590 (2)	0.0501 (9)	
H4N	0.541 (5)	0.458 (4)	0.183 (3)	0.060*	
N5	0.6762 (5)	0.6224 (3)	0.1738 (2)	0.0468 (8)	
N6	0.7780 (5)	0.8026 (3)	0.12079 (19)	0.0473 (8)	
H6N	0.804 (6)	0.847 (4)	0.1678 (15)	0.057*	
C10	0.8011 (5)	0.4900 (4)	0.3167 (3)	0.0461 (10)	
C11	0.9365 (6)	0.6028 (4)	0.3554 (3)	0.0593 (12)	
H11	1.0191	0.6482	0.3273	0.071*	
C12	0.9484 (6)	0.6473 (4)	0.4349 (3)	0.0605 (12)	
H12	1.0383	0.7233	0.4607	0.073*	
C13	0.8276 (6)	0.5797 (4)	0.4762 (3)	0.0540 (11)	
C14	0.6935 (6)	0.4666 (5)	0.4392 (3)	0.0611 (12)	
H14	0.6137	0.4207	0.4682	0.073*	
C15	0.6792 (6)	0.4226 (4)	0.3595 (3)	0.0566 (11)	
H15	0.5875	0.3474	0.3339	0.068*	
C16	0.7281 (5)	0.6717 (4)	0.1146 (2)	0.0438 (9)	
C17	0.8171 (6)	0.8523 (5)	0.0550 (3)	0.0575 (12)	
C18	0.8040 (7)	0.7499 (5)	−0.0134 (3)	0.0652 (13)	
H18A	0.9219	0.7657	−0.0308	0.078*	
H18B	0.7080	0.7488	−0.0586	0.078*	
O7	0.9751 (5)	0.1699 (4)	0.1821 (3)	0.0766 (10)	
H71	0.880 (5)	0.191 (6)	0.182 (4)	0.092*	
H72	0.933 (8)	0.112 (4)	0.143 (3)	0.092*	
O8	0.3062 (6)	0.4099 (6)	0.2195 (4)	0.1111 (16)	
H81	0.195 (4)	0.390 (7)	0.199 (4)	0.133*	
H82	0.303 (12)	0.472 (5)	0.250 (4)	0.133*	

### Atomic displacement parameters (Å^2^)

**Table:**

	*U*^11^	*U*^22^	*U*^33^	*U*^12^	*U*^13^	*U*^23^
Cl1	0.0697 (8)	0.0852 (9)	0.0517 (7)	0.0274 (7)	0.0175 (6)	−0.0042 (6)
S1	0.0486 (6)	0.0477 (6)	0.0378 (6)	0.0020 (5)	0.0078 (4)	0.0023 (5)
S2	0.0572 (7)	0.0670 (8)	0.0452 (6)	0.0120 (6)	0.0114 (5)	−0.0085 (5)
O1	0.0601 (19)	0.057 (2)	0.059 (2)	−0.0078 (15)	0.0119 (15)	−0.0071 (15)
O2	0.070 (2)	0.072 (2)	0.0449 (18)	0.0156 (17)	0.0139 (15)	0.0183 (16)
O3	0.059 (2)	0.068 (2)	0.088 (3)	0.0044 (18)	0.0237 (18)	0.0185 (19)
N1	0.049 (2)	0.039 (2)	0.049 (2)	0.0029 (16)	0.0122 (17)	−0.0008 (15)
N2	0.0460 (19)	0.046 (2)	0.0420 (19)	0.0047 (16)	0.0111 (16)	−0.0001 (15)
N3	0.0411 (18)	0.049 (2)	0.042 (2)	0.0083 (16)	0.0061 (15)	0.0010 (16)
C1	0.039 (2)	0.041 (2)	0.044 (2)	0.0106 (17)	0.0078 (17)	0.0082 (18)
C2	0.057 (3)	0.042 (2)	0.053 (3)	0.007 (2)	0.015 (2)	0.014 (2)
C3	0.058 (3)	0.041 (2)	0.055 (3)	0.011 (2)	0.018 (2)	0.002 (2)
C4	0.046 (2)	0.056 (3)	0.043 (2)	0.019 (2)	0.0111 (18)	0.002 (2)
C5	0.079 (3)	0.058 (3)	0.052 (3)	0.013 (2)	0.030 (2)	0.017 (2)
C6	0.077 (3)	0.037 (2)	0.056 (3)	0.006 (2)	0.025 (2)	0.010 (2)
C7	0.038 (2)	0.046 (2)	0.039 (2)	0.0147 (18)	0.0014 (17)	0.0022 (18)
C8	0.044 (2)	0.060 (3)	0.057 (3)	0.020 (2)	0.011 (2)	0.014 (2)
C9	0.056 (3)	0.080 (3)	0.052 (3)	0.027 (2)	0.021 (2)	0.013 (2)
Cl2	0.0968 (10)	0.0802 (10)	0.0634 (8)	0.0332 (8)	0.0091 (7)	0.0040 (7)
S3	0.0483 (6)	0.0431 (6)	0.0643 (7)	0.0136 (5)	0.0182 (5)	0.0104 (5)
S4	0.0632 (7)	0.0641 (8)	0.0448 (6)	0.0112 (6)	0.0121 (5)	−0.0066 (5)
O4	0.0518 (18)	0.073 (2)	0.084 (2)	0.0203 (16)	0.0285 (17)	0.0204 (19)
O5	0.084 (2)	0.0379 (17)	0.087 (3)	0.0158 (16)	0.0302 (19)	0.0047 (16)
O6	0.097 (3)	0.066 (2)	0.071 (2)	0.021 (2)	0.028 (2)	0.0261 (19)
N4	0.052 (2)	0.041 (2)	0.050 (2)	0.0078 (17)	0.0055 (16)	−0.0003 (16)
N5	0.052 (2)	0.044 (2)	0.042 (2)	0.0137 (16)	0.0069 (16)	0.0037 (16)
N6	0.057 (2)	0.051 (2)	0.0315 (18)	0.0168 (17)	0.0033 (16)	0.0056 (16)
C10	0.038 (2)	0.039 (2)	0.058 (3)	0.0082 (18)	0.0031 (18)	0.0137 (19)
C11	0.056 (3)	0.050 (3)	0.061 (3)	−0.003 (2)	0.008 (2)	0.020 (2)
C12	0.059 (3)	0.039 (3)	0.068 (3)	−0.001 (2)	−0.006 (2)	0.013 (2)
C13	0.057 (3)	0.047 (3)	0.058 (3)	0.021 (2)	0.001 (2)	0.014 (2)
C14	0.057 (3)	0.059 (3)	0.063 (3)	0.007 (2)	0.017 (2)	0.015 (2)
C15	0.043 (2)	0.047 (3)	0.072 (3)	0.0000 (19)	0.016 (2)	0.006 (2)
C16	0.038 (2)	0.050 (3)	0.040 (2)	0.0146 (18)	0.0011 (17)	0.0015 (19)
C17	0.052 (3)	0.072 (3)	0.047 (3)	0.016 (2)	0.010 (2)	0.011 (2)
C18	0.072 (3)	0.073 (3)	0.049 (3)	0.016 (3)	0.018 (2)	0.011 (2)
O7	0.077 (3)	0.065 (2)	0.093 (3)	0.030 (2)	0.013 (2)	0.0167 (19)
O8	0.056 (2)	0.146 (5)	0.131 (4)	0.030 (3)	0.029 (3)	0.013 (3)

### Geometric parameters (Å, º)

**Table:**

Cl1—C4	1.744 (4)	S3—O5	1.441 (3)
S1—O1	1.422 (3)	S3—N4	1.649 (4)
S1—O2	1.438 (3)	S3—C10	1.759 (5)
S1—N1	1.644 (4)	S4—C16	1.746 (4)
S1—C1	1.770 (4)	S4—C18	1.812 (5)
S2—C7	1.741 (4)	O6—C17	1.218 (6)
S2—C9	1.808 (5)	N4—N5	1.442 (5)
O3—C8	1.202 (5)	N4—H4N	0.850 (19)
N1—N2	1.440 (5)	N5—C16	1.276 (5)
N1—H1N	0.842 (19)	N6—C17	1.353 (6)
N2—C7	1.285 (5)	N6—C16	1.381 (5)
N3—C7	1.370 (5)	N6—H6N	0.851 (19)
N3—C8	1.374 (5)	C10—C11	1.389 (6)
N3—H3N	0.851 (19)	C10—C15	1.390 (6)
C1—C6	1.377 (6)	C11—C12	1.369 (7)
C1—C2	1.385 (6)	C11—H11	0.9300
C2—C3	1.389 (6)	C12—C13	1.368 (6)
C2—H2	0.9300	C12—H12	0.9300
C3—C4	1.366 (6)	C13—C14	1.380 (6)
C3—H3	0.9300	C14—C15	1.368 (6)
C4—C5	1.379 (6)	C14—H14	0.9300
C5—C6	1.381 (6)	C15—H15	0.9300
C5—H5	0.9300	C17—C18	1.498 (7)
C6—H6	0.9300	C18—H18A	0.9700
C8—C9	1.503 (6)	C18—H18B	0.9700
C9—H9A	0.9700	O7—H71	0.82 (2)
C9—H9B	0.9700	O7—H72	0.82 (2)
Cl2—C13	1.737 (5)	O8—H81	0.82 (2)
S3—O4	1.426 (3)	O8—H82	0.83 (2)
			
O1—S1—O2	120.4 (2)	O4—S3—N4	107.3 (2)
O1—S1—N1	105.18 (19)	O5—S3—N4	103.2 (2)
O2—S1—N1	105.77 (19)	O4—S3—C10	107.8 (2)
O1—S1—C1	108.45 (19)	O5—S3—C10	108.6 (2)
O2—S1—C1	107.88 (19)	N4—S3—C10	109.61 (18)
N1—S1—C1	108.64 (18)	C16—S4—C18	91.4 (2)
C7—S2—C9	91.4 (2)	N5—N4—S3	112.3 (3)
N2—N1—S1	113.6 (3)	N5—N4—H4N	106 (3)
N2—N1—H1N	106 (3)	S3—N4—H4N	97 (3)
S1—N1—H1N	107 (3)	C16—N5—N4	112.6 (3)
C7—N2—N1	111.2 (3)	C17—N6—C16	117.8 (4)
C7—N3—C8	117.6 (4)	C17—N6—H6N	124 (3)
C7—N3—H3N	122 (3)	C16—N6—H6N	117 (3)
C8—N3—H3N	119 (3)	C11—C10—C15	119.4 (4)
C6—C1—C2	120.4 (4)	C11—C10—S3	120.0 (3)
C6—C1—S1	119.8 (3)	C15—C10—S3	120.6 (3)
C2—C1—S1	119.8 (3)	C12—C11—C10	120.1 (4)
C1—C2—C3	119.6 (4)	C12—C11—H11	119.9
C1—C2—H2	120.2	C10—C11—H11	119.9
C3—C2—H2	120.2	C13—C12—C11	119.7 (4)
C4—C3—C2	119.0 (4)	C13—C12—H12	120.1
C4—C3—H3	120.5	C11—C12—H12	120.1
C2—C3—H3	120.5	C12—C13—C14	121.2 (5)
C3—C4—C5	122.1 (4)	C12—C13—Cl2	119.4 (4)
C3—C4—Cl1	118.6 (3)	C14—C13—Cl2	119.4 (4)
C5—C4—Cl1	119.2 (3)	C15—C14—C13	119.4 (4)
C4—C5—C6	118.7 (4)	C15—C14—H14	120.3
C4—C5—H5	120.7	C13—C14—H14	120.3
C6—C5—H5	120.7	C14—C15—C10	120.3 (4)
C1—C6—C5	120.2 (4)	C14—C15—H15	119.9
C1—C6—H6	119.9	C10—C15—H15	119.9
C5—C6—H6	119.9	N5—C16—N6	118.7 (4)
N2—C7—N3	119.3 (4)	N5—C16—S4	129.9 (3)
N2—C7—S2	128.6 (3)	N6—C16—S4	111.3 (3)
N3—C7—S2	112.1 (3)	O6—C17—N6	124.2 (4)
O3—C8—N3	123.9 (4)	O6—C17—C18	124.6 (4)
O3—C8—C9	125.7 (4)	N6—C17—C18	111.2 (4)
N3—C8—C9	110.4 (4)	C17—C18—S4	108.0 (3)
C8—C9—S2	108.4 (3)	C17—C18—H18A	110.1
C8—C9—H9A	110.0	S4—C18—H18A	110.1
S2—C9—H9A	110.0	C17—C18—H18B	110.1
C8—C9—H9B	110.0	S4—C18—H18B	110.1
S2—C9—H9B	110.0	H18A—C18—H18B	108.4
H9A—C9—H9B	108.4	H71—O7—H72	98 (6)
O4—S3—O5	119.9 (2)	H81—O8—H82	94 (7)
			
O1—S1—N1—N2	172.9 (3)	O4—S3—N4—N5	−59.2 (3)
O2—S1—N1—N2	−58.6 (3)	O5—S3—N4—N5	173.2 (3)
C1—S1—N1—N2	57.0 (3)	C10—S3—N4—N5	57.6 (3)
S1—N1—N2—C7	111.8 (3)	S3—N4—N5—C16	109.7 (3)
O1—S1—C1—C6	−22.2 (4)	O4—S3—C10—C11	23.0 (4)
O2—S1—C1—C6	−154.2 (4)	O5—S3—C10—C11	154.4 (3)
N1—S1—C1—C6	91.6 (4)	N4—S3—C10—C11	−93.5 (4)
O1—S1—C1—C2	158.0 (3)	O4—S3—C10—C15	−157.6 (3)
O2—S1—C1—C2	26.0 (4)	O5—S3—C10—C15	−26.3 (4)
N1—S1—C1—C2	−88.2 (4)	N4—S3—C10—C15	85.8 (4)
C6—C1—C2—C3	−1.2 (6)	C15—C10—C11—C12	−0.4 (7)
S1—C1—C2—C3	178.6 (3)	S3—C10—C11—C12	179.0 (4)
C1—C2—C3—C4	−0.1 (6)	C10—C11—C12—C13	0.6 (7)
C2—C3—C4—C5	1.2 (7)	C11—C12—C13—C14	0.2 (7)
C2—C3—C4—Cl1	−177.7 (3)	C11—C12—C13—Cl2	−179.1 (3)
C3—C4—C5—C6	−1.1 (7)	C12—C13—C14—C15	−1.2 (7)
Cl1—C4—C5—C6	177.9 (4)	Cl2—C13—C14—C15	178.1 (4)
C2—C1—C6—C5	1.4 (7)	C13—C14—C15—C10	1.3 (7)
S1—C1—C6—C5	−178.4 (4)	C11—C10—C15—C14	−0.6 (7)
C4—C5—C6—C1	−0.3 (7)	S3—C10—C15—C14	−179.9 (4)
N1—N2—C7—N3	−176.1 (3)	N4—N5—C16—N6	−179.6 (3)
N1—N2—C7—S2	5.0 (5)	N4—N5—C16—S4	0.8 (5)
C8—N3—C7—N2	178.3 (4)	C17—N6—C16—N5	−174.4 (4)
C8—N3—C7—S2	−2.6 (5)	C17—N6—C16—S4	5.3 (5)
C9—S2—C7—N2	−179.0 (4)	C18—S4—C16—N5	174.5 (4)
C9—S2—C7—N3	2.0 (3)	C18—S4—C16—N6	−5.1 (3)
C7—N3—C8—O3	−178.6 (4)	C16—N6—C17—O6	177.6 (4)
C7—N3—C8—C9	1.6 (5)	C16—N6—C17—C18	−2.1 (5)
O3—C8—C9—S2	−179.7 (4)	O6—C17—C18—S4	178.5 (4)
N3—C8—C9—S2	0.0 (5)	N6—C17—C18—S4	−1.8 (5)
C7—S2—C9—C8	−1.1 (3)	C16—S4—C18—C17	3.9 (3)

### Hydrogen-bond geometry (Å, º)

**Table:**

*D*—H···*A*	*D*—H	H···*A*	*D*···*A*	*D*—H···*A*
N1—H1*N*···O7^i^	0.84 (2)	2.07 (2)	2.900 (6)	168 (4)
N3—H3*N*···N5^ii^	0.85 (2)	2.07 (2)	2.895 (5)	162 (4)
C9—H9*B*···O2^ii^	0.97	2.42	3.236 (6)	141
N4—H4*N*···O8	0.85 (2)	1.95 (2)	2.788 (6)	168 (4)
N6—H6*N*···N2^iii^	0.85 (2)	1.97 (2)	2.808 (5)	170 (4)
C15—H15···O1^iv^	0.93	2.55	3.355 (5)	145
O7—H71···O5	0.82 (2)	2.08 (3)	2.868 (5)	162 (6)
O7—H72···O6^iv^	0.82 (2)	1.99 (2)	2.810 (5)	174 (6)
O8—H81···O4^ii^	0.82 (2)	2.35 (6)	2.987 (6)	136 (7)
O8—H81···O7^ii^	0.82 (2)	2.50 (7)	3.034 (7)	124 (7)
O8—H82···O3^iii^	0.83 (2)	2.37 (3)	3.159 (7)	159 (8)

Symmetry codes: (i) *x*−1, *y*+1, *z*; (ii) *x*−1, *y*, *z*; (iii) *x*+1, *y*, *z*; (iv) *x*, *y*−1, *z*.
